# Nutritional Potential and Protective Effects of *Colocassia esculenta* Flowers Against 2,4‐Dinitrophenyl Hydrazine (DNPH) Induced Anemia *Wistar* Strain Rats

**DOI:** 10.1002/fsn3.70419

**Published:** 2025-06-13

**Authors:** Josée Rebecca Nombo, Armel Junior Mbock, Jules Christophe Koule Manz, Tene Stephano Tambo, Misse Jethro Roland Ngangue Ekwalla, Valery Jean François Nsoga, Mathieu Ndomou

**Affiliations:** ^1^ Laboratory of Biochemistry, Faculty of Science University of Douala Douala Cameroon; ^2^ Laboratory of Animal Biology, Faculty of Sciences University of Douala Douala Cameroon; ^3^ Research Unit of Biochemistry of Medicinal Plants, Food Science and Nutrition, Department of Biochemistry, Faculty of Science University of Dschang Dschang Cameroon

**Keywords:** anemia, *Colocassia esculenta*, DNPH, nutritional potential, oxidative stress

## Abstract

This study aimed to assess the nutritional value and effect of *Colocasia esculenta* flowers on induced anemia in *Wistar* rats. Proximate composition and minerals analysis were performed using AOAC standard methods. Twenty‐five rats were randomly divided into five groups. Rats in Group 1 were healthy and non‐anemic. In Group 2, the rats were anemic and untreated. Anemic rats in Group 3 were given 5 mg/Kg of a reference drug (Apfer) against anemia. Rats in Groups 4 and 5 were anemic and fed with *C. esculenta* flower powder (5% substitution) harvested in Dschang and Yabassi. Anemia was induced in rats using 2,4‐Dinitrophenyl hydrazine. After 23 days of treatment, some hematological and biochemical parameters as well as indicators of oxidative stress were evaluated. The results show that potassium, calcium, and phosphate were the main minerals present in *C. esculenta* flowers. Concerning proteins (21.29% ± 1.13%) and lipids content (8.99% ± 0.72%), flowers harvested in Dschang were the most nutritive. Calcium was more available in flowers harvested in Dschang. These flowers are the potential sources of carotenoids with around 2201.33 and 2535.04 mg/100 g, harvested in Dschang and Yabassi, respectively. The main metabolites identified were alkaloids, quinones, anthocyanins, triterpenes, and saponins, and this profile was not affected by the locality. *C. esculenta* flowers significantly (*p <* 0.05) increased hematocrit, hemoglobin, and red blood cells in Groups 4 and 5 compared to Group 2. Supplementation of *C. esculenta* flower significantly (*p <* 0.05) increased lipid profile in Groups 4 and 5 when compared to Groups 2 and 3. Also, the activity of superoxide dismutase (SOD) and catalase (CAT) significantly (*p* < 0.05) increased in Groups 4 and 5 compared to Groups 2 and 3. Consumption of *C. esculenta* flowers could therefore help to prevent and fight against hemolytic anemia and oxidative stress.

## Graphical abstract

The nutritional properties of *C. esculenta* have been demonstrated. Consumption of *C. esculenta* reduces anemic disorder in Wistar rats. *C. esclenta* harvested in Dschang has the best anti‐anemic potential.

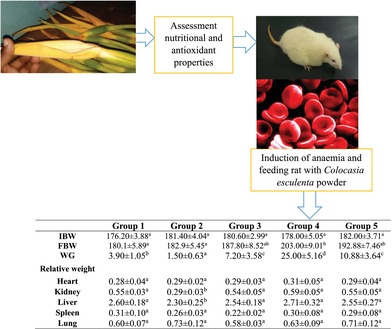

## 1 Introduction

Anemia is the most prevalent hemolytic disorder in developing countries. According to Chandrakumari et al. (2019), anemia occurs when hemoglobin concentration is less than 12 mg/dL in women and 13 mg/dL in men. Hemolytic anemia is less common than iron deficiency anemia. Hemoglobin levels in the blood are substantially reduced by iron deficiency, which significantly affects the blood's capacity to carry oxygen to the tissues. In most cases, iron deficiency is due to low iron intakes. It could also arise as a consequence of increased iron requirements during growth and pregnancy. According to the World Health Organization [WHO] (2017), anemia is a public health issue that reduce growth, inhibits economic and social welfare, and raises infection‐related morbidity. The WHO estimates shows that around 40% of pregnant women and more than 42% of children under the age of five worldwide are affected by anemia (WHO 2017). Also, one‐third of women (33%) of reproductive age (15 to 49 years old) are anemic (Kunnath et al. 2021). The incidence of anemia is higher in developing countries due to poor nutrition, low socioeconomic status, high prevalence of blood parasites including plasmodium, trypanosomes, and helminthic infections (WHO 2017). In developing countries such as Cameroon, the risks of morbidity and mortality due the anemia is increasing (Zangenehn et al. 2017). Surveys made by the Cameroon's Ministry of Public Health showed that the prevalence of anemia was 57% in children aged 6 to 59 months and 39.7% in women of childbearing age (Ministère de la Santé Publique au Cameroun–Organisation Mondiale de la Santé 2019). Understanding the causes of anemia in developing countries is a stepping stone to its effective management. Ogukwe et al. (2017) have shown a relationship between hemolytic anemia and oxidative stress, which appears to be one of the causes of the disease via glucose 6‐phosphate dehydrogenase deficiency in the erythrocyte membrane. Apoptosis and hemolysis of erythrocytes are mainly due to oxidative stress and aging (Gammal et al. 2023). Currently, many practices are used to fight against anemia, including iron supplementation or fortification, diversification of food sources, awareness of vulnerable individuals, blood transfusions, and injection of erythropoietin. These methods are expensive and not always available. They also have significant side effects (Ousaaid et al. 2022). The use of plants is an alternative within everyone's reach. The FAO suggests that the diversity of food plants found in African countries could be a source of solution. These plants have beneficial properties and are used in the management of various ailments, thanks to some useful secondary metabolites that can protect humans against diseases (Massohe et al. 2023). *Colocasia esculenta* or taro flowers, locally known as “Achu” is an example of these plants. It is a monocotyledonous plant of the *Araceae* family. Tubers and leaves of *C. esculenta* are the main parts used for nutritional and therapeutic purposes. The leaves are scarcely used as food. In some villages of the West and Littoral regions of Cameroon, leaves and tubers of “taro” are introduced in soups to enhance taste. They could also have good therapeutic properties but are unfortunately less used, neglected compared to tubers and leaves. *C. esculenta* flowers have appreciated amounts of proteins, fibers, carbohydrates, potassium, calcium, magnesium, and many classes of metabolites like phenols, tannins, terpenoids, sterols, quinones, glycosides, and saponins (Ogukwe et al. 2017). Some studies have mentioned the antihyperglycemic and antioxidant potential of extracts from these flowers (Kumawat et al. 2010; Yadav et al. 2017). Elsewhere, Okechukwu et al. (2020) found that cocoyam inflorescence has an appreciable amount of proteins, minerals, fat, fiber, carbohydrates, vitamins A and C, and low levels of toxicants. Ouoba et al. (2022) evaluated the nutritional potential of the leaves of five varieties of *C. esculenta* grown in Burkina Faso and found that young leaves are of nutritional interest in view of their nutrient contents. A survey conducted by Kashyap et al. (2023) on the nutritional and therapeutic benefits of *C. esculenta* leaves revealed their medicinal, nutritional, and physiological benefits, including antioxidant, anti‐inflammatory, analgesic, anti‐lipid peroxidation, anti‐diabetic, and anti‐hepatotoxic properties. However, to the best of our knowledge, no study has been done on the anti‐anemic properties of *C. esculenta* flowers. As part of the valorization of this endogenous under‐exploited product, we evaluated the anti‐anemic potential and nutritional properties of the flower of *C. esculenta* in *Wistar* strain rats.

## 2 Methodology

### 2.1 Plant Material

The fresh flowers of *C. esculenta* were harvested in Yabassi (4°27′16″ North latitude and 9°57′56″ East longitude) and Dschang (5°27′00″ latitude North and 10°04′00″ East longitude) respectively from Littoral and West regions of Cameroon. The leaves were sorted, washed, cut, and dried at 45°C during 48 h in an oven (BINDER‐78532). The dried leaves were ground using a mechanical mixer (SWISS LINE SWITZERLAND UNIVERSAL MIXER SW‐1354‐P) and the powders obtained were stored at −20°C freezer (HISENSE CONGE 590 HS590) for further analysis.

### 2.2 Experimental Animals

The animals used in this study were *Wistar* strain albino rats with an average weight of 179.24 ± 4.97 g. The rats were placed in appropriated polystyrene cages and they were fed ad libitum. The experiments were carried out in accordance with internationally and nationally accepted guidelines for studies on experimental animals. The University of Douala Ethical Committee approved this work (No. 2304 CEI‐Udo/05/2020/T).

### 2.3 Methods

#### 2.3.1 Proximate Composition

The water content was obtained by drying the samples at 105°C until a constant weight according to Association of the Official Analysis Chemists [AOAC] (1990) standard method. Lipids were extracted by Soxhlet's method using hexane as solvent. Proteins were determined by conversion into nitrogen using the Kjeldahl method. For determination of ash content, the sample was incinerated for 20 h at 500°C. The standard method of AOAC (1990) allows us to quantify fiber content, while total carbohydrates were obtained after subtracting moisture, protein, lipid, and ash contents according to Equation (1) (AOAC 1990):

(1) $$\text{Total carbohydrate}=100–\left(\text{moisture}+\text{protein}+\text{lipid}+\mathrm{ash}\right)$$

#### 2.3.2 Mineral Analysis

An amount of 2.5 g of the sample was introduced in a Pyrex beaker and incinerated for 20 h at 500°C in a muffle furnace. The ashes obtained from this process were transferred in 100 mL beakers and the remaining organic matter digested with 10 mL of concentrated nitric acid. The digestion was conducted at 95°C using a water bath for 30 min. The digested solution was filtered into a 100 mL conical flask and the volume was completed with distilled water to the top. This solution was used to quantify the different minerals (calcium, potassium, magnesium, sodium, iron, zinc, copper and manganese) at their different wavelengths using atomic absorption spectrophotometry (PerkinElmer Atomic Absorption Spectrometer Pinnacle 900 T, Perkin Elma, USA). The same solution was used for the quantification of phosphorus using a spectrophotometer at 860 nm (PerkinElmer, Norwalk, CT, USA). Total carotenoids content was obtained by the photometry method, reported by Dongho et al. (2014) using an Ichek Photometre (TM carotène; BioAnalyt GmbH, Teltow, Germany).

#### 2.3.3 Preparation of Extracts and Phytochemical Screening (Qualitative and Quantitative)

The leaf powder of *C. esculenta* was macerated in water for 72 h and then filtered using Whatman No. 4 filter paper. Filtrates were concentrated in a rotary evaporator (BÜCHI ROTAVAPOR R‐205) at 40°C. The extract obtained was dried in an oven at 40°C and then stored in a clean and dry bottle at −20°C freezer (HISENSE CONGE 590 HS590) until use (Bamlaku and Gedefaw 2020). A phytochemical screening was carried out on *C. esculenta* aqueous powder extract to check the presence of secondary metabolites (Bankole et al. 2016).

For quantitative assay, the total polyphenol contents were estimated by colorimetric method using folin–Ciocalteu as reagent and gallic acid as standard or reference molecule (Jaradat et al. 2020). The amount of phenols obtained was expressed in milliequivalent gram of gallic acid per mg of extract (mg EAG/g of extract). Flavonoid content was assayed using the methodology described by Jaradat et al. (2020). This colorimetric method required AlCl_3_, NaNO_2_, and NaOH as reagents and quercetin as reference molecule. The amount of flavonoids obtained was expressed in milliequivalent gram of quercetin per mg of extract (mg EQ/g of extract) according to the quercetin standard curve. The tannin contents were determined by the vanillin method in an acid medium, as described by Mignanwandé et al. (2020). Tannin contents were determined using a linear calibration curve with catechin as reference, and the results were expressed in milliequivalent gram of catechin per mg of extract (mg EC/g of extract). All samples were read in triplicate against blank.

#### 2.3.4 Experimental Design and Induction of Hemolytic Anemia

Twenty‐five rats were divided into five groups: Group 1: non‐anemic rats fed a standard laboratory diet (RSL); Group 2: untreated anemic rats fed RSL; Group 3: anemic rats fed RSL supplemented with a reference drug (Apfer) (5 mg/kg); Group 4: anemic rats fed RSL supplemented with 5% *C. esculenta* powder harvested in Dschang; Group 5: anemic rats fed RSL supplemented with 5% *C. esculenta* powder harvested in Yabassi. The substitution percentage was selected according to the work done by Doungue et al. (2020) to avoid a toxicity effect. Anemia was induced by intraperitoneal injection of a solution of 2,4‐dinitrophenylhydrazine at a concentration of 60 mg/kg for two consecutive days. Rats which expressed a drop in hemoglobin level of more than 30% were considered anemic (Anupam et al. 2014). To achieve this, an automatic hemoglobin counter and strips were used. Table 1 shows the composition of the diet according to the modified protocol of Aboubacar et al. (2020).

**TABLE 1:** Diet composition.

Ingredients (%)	Group 1	Group 2	Group 3	Group 4	Group 5
Corn flour	48.3	48.3	48.3	48.3	48.3
Soy flour	13.7	13.7	13.7	8.7	8.7
Remoulage	8.6	8.6	8.6	8.6	8.6
Palm kernel flour	6.9	6.9	6.9	6.9	6.9
Fishmeal	6.9	6.9	6.9	6.9	6.9
Peanut flour	5.2	5.2	5.2	5.2	5.2
Concentrate	3.45	3.45	3.45	3.45	3.45
Wheat brand	3.45	3.45	3.45	3.45	3.45
Taro flower flour	0	0	0	5	5
Vitamin complex	1	1	1	1	1
Oyster shell meal	1	1	1	1	1
Salt	1	1	1	1	1
Apfer	0	0	0.085	0	0

*Note:* Group 1, non‐anemic rats fed RSL; Group 2, RSL‐fed untreated anemic rats; Group 3, anemic rats fed RSL and treated with Apfer (5 mg/kg); Group 4, anemic rats fed RSL supplemented with 5% *Colocasia esculenta* flower powder harvested in Dschang; Group 5, anemic rats fed RSL supplemented with 5% *C. esculenta* flower powder harvested in Yabassi.

#### 2.3.5 Collection of Blood Samples

Blood was collected on Days 0, 2, 9, 16, and 23. In fact, blood was collected by cardiac puncture from the left ventricles. The animals were anesthetized by subcutaneous injection of the Diazepam‐ketamine mixture using an insulin pin (0.2 mL/100 g of body weight and 0.1 mL/100 g of body weight respectively). After 23 days of treatment, the rats were fasted for 24 h (to avoid any influence from feeding) and sacrificed. Blood from each rat was collected immediately in a tubes without EDTA and EDTA tubes respectively. Sera obtained from dry tubes were stored at −20°C freezer (HISENSE CONGE 590 HS590) for the assay of biochemical parameters. The plasma obtained from EDTA tubes was used for the determination of some hematological parameters of the rats from Days D0, D2, D9, D16, and D23. Rats' hearts, livers, lungs, kidneys, and spleen were subsequently removed, rinsed with normal saline, and then weighed.

#### 2.3.6 Analysis of Hematological Parameters

Red and white blood cells, hemoglobin, hematocrit, lymphocytes, blood platelets, mean corpuscular volume, and hemoglobin content and concentration were determined using an Automated Hematological Counter (Nihon Kohden ME6411K).

#### 2.3.7 Analysis of Serum Biochemical Parameters and Stress Markers

The total lipid profile of serum (total cholesterol, HDL, LDL and triglycerides), total protein, urea, alkaline phosphatase (PAL), Gamma glutamyl transferase (γGT), creatinine, and activities of ASAT (aspartate aminotransferase) and ALAT (alanine aminotransferase) were assayed by colorimetric enzymatic methods using the corresponding kit (SGM Italia, Italy) according to the methods cited by Nchoutpouen et al. (2020). Levels of thiobarbituric acid reactive substances (TBARS), reduced glutathione (GSH), catalase (CAT) and superoxide dismutase (SOD) activities were assessed by spectrophotometry (Perkin Elmer, Norwalk CT, USA) using the method reported by Aloud (2019). The level of reduced glutathione (GSH) was determined by a method described by Ellman (1959). In test tubes, 20 μL of serum or liver or kidney homogenates and 3 mL of Ellman's reagent were introduced. Absorbance was read at 412 nm against blank. Catalase activity was quantified by mixing 50 μL of homogenate with 750 μL of 0.1 mM phosphate buffer pH 7.5 and 200 μL of H_2_O_2_ (50 mM). The mixture was pre‐incubated for 1 min at 25°C before the addition of 2 mL K_2_Cr_2_O_2_, then heated at 100°C for 10 min, followed by a spectrophotometer reading at 570 nm. For SOD, 134 μL of serum was mixed with 1666 μL of 0.05 M carbonate buffer pH 10.2 and 200 μL of 0.3 mM adrenaline before the spectrophotometer reading at 480 nm.

### 2.4 Statistical Analysis

The data from the various tests were analyzed by the Stat graphic Centurion Version 17.1.8 software and presented as mean ± standard deviation. One‐way ordered analysis of variance (ANOVA) was used to compare group means. Fisher's PLSD (Protected Least Significant Difference) (post hoc comparison test) was used for comparisons when the ANOVA *p*‐value (*p* ≤ 0.05) was significant.

## 3 Results and Discussion

### 3.1 Proximate Composition

The proximate chemical composition of the *C. esculenta* flowers is presented in Table 2. Moisture appeared to be the major component of this flower, with content laying between 85.33% and 86%. The content was not affected (*p >* 0.05) by the collection sites. Our results were lower than those found by Kouamé et al. (2021) on the leaves of the same plant (93.70%). Leaves are the plant's main respiratory organ (Oulai et al. 2019). The water content is an important parameter for the preservation of foodstuffs. The higher the water content, the more sensitive to degradation the plant is (Manz et al. 2023). The mean value of protein content (19.48% DM) varied significantly (*p <* 0.05) between locations. Geographical origin, climatic conditions, transport, and storage conditions can influence the chemical composition (Oulai et al. 2019). These flowers could be used as a food supplement in the fight against protein malnutrition and constitute an alternative to solving health problems linked to the consumption of meat. Given its richness in protein, *C. esculenta* flowers could be used to compensate for protein imbalances during weaning periods and ensure nutritional balance for individuals consuming it. Also, lipid content (8.15% DM) varies significantly (*p <* 0.05) and was lower than that found by Ogukwe et al. (2017) in the flowers of *C. esculenta* (14.13%) but was higher than that found by Ouoba et al. (2022) on the variety BF/CO/04 of *C. esculenta* (0.28%). The method of extraction and place of harvest could explain this result. The flowers of *C. esculenta* could enrich the repertoire of low‐calorie foods requested by obese patients. The flowers of *C. esculenta* are a good source, as evidenced by their high total carbohydrate and fiber contents (Okechukwu et al. 2020). The Recommended Dietary Allowances of fiber are 22–23 g of fiber for every 1000 kcal of diet (Mohammed et al. 2022). Consuming 72.21 g of *C. esculenta* flower powder harvested in Yabassi could cover 100% of the fiber intake recommended by the WHO. These flower powders could be recommended for people with digestion problems, particularly for people suffering from constipation. Fibers are recognized for their crucial role in improving intestinal transit, their lipid‐lowering, and hypoglycemic properties (Tuem et al. 2022). The ash content of *C. esculenta* flowers is, in particular, 7.33% DM and 10.18% DM, and also varied significantly (*p* < 0.05) between the two collection locations. These results are higher than those obtained by Ogukwe et al. (2017) on the flowers of *Xanthosoma sagittifolium* (6.23%). The quantity of the minerals present in food samples can be assessed through ash content (Dama et al. 2021).

**TABLE 2:** Proximate composition and mineral content of *C. esculenta* flowers.

Parameters	Nutritional value of flowers from Dschang	Nutritional value of flowers from Yabassi
Moisture (% WW)	85.33 ± 1.05^a^	86.00 ± 0.51^a^
Crude protein (% DM)	21.29 ± 1.13^b^	17.67 ± 0.86^a^
Lipid (% DM)	8.99 ± 0.72^b^	7.31 ± 0.53^a^
Carbohydrate (% DM)	62.39 ± 0.83^a^	64.83 ± 0.26^b^
Crude fiber (% DM)	27.40 ± 1.70^a^	31.85 ± 0.96^b^
Ash content (% DM)	7.33 ± 1.15^a^	10.18 ± 0.58^b^
Mg (mg/100 g DM)	982.37 ± 24.02^b^	194.10 ± 19.68^a^
Ca (mg/100 g DM)	1936.00 ± 21.79^a^	5202.59 ± 12.40^b^
P (mg/100 g DM)	576.98 ± 44.33^b^	487.90 ± 23.17^a^
Ca/P	3.35 ± 0.14^a^	10.66 ± 1.36^b^
Na (mg/100 g DM)	356.73 ± 19.90^b^	212.53 ± 31.60^a^
K (mg/100 g DM)	2439.47 ± 25.03^a^	2415.41 ± 42.34^a^
Na/K	0.14 ± 0.03^b^	0.08 ± 0.00^a^
Fe (mg/100 g DM)	17.16 ± 1.50^a^	29.33 ± 0.08^b^
Zn (mg/100 g DM)	0.44 ± 0.33^a^	1.86 ± 0.44^b^
Mn (mg/100 g DM)	0.36 ± 0.09^a^	0.51 ± 0.13^a^
TCR (μg/100 g DM)	2201.33 ± 19.36^a^	2535.04 ± 8,53^b^

*Note:* The values are the mean ± standard deviation (*n* = 3). Values in line with different letters are significantly (*p* < 0.05) different.

Abbreviations: DM, dry matter; Flower 1, *Colocasia esculenta* flower harvested in the city of Dschang; Flower 2, *C. esculenta* flower harvested in the city of Yabassi; TCR, total carotenoids; WW, wet weight.

### 3.2 Minerals Content

The minerals composition is shown in Table 2. Contents of magnesium, phosphorus, calcium, sodium, iron, and zinc were significantly (*p <* 0.05) influenced by the collection sites. However, potassium content did not significantly (*p >* 0.05) vary between the two collection sites. This result could be explained by the difference in geographical origin of the collection sites (Oulai et al. 2019). The magnesium contents obtained by Okechukwu et al. (2020) and Kashyap et al. (2023) on the flowers and leaves of the black green varieties of *C. esculenta* are lower than those found in this study. Magnesium is involved in the regulatory action of insulin and body temperature, muscle contraction, energy metabolism, and protein synthesis (Kacem et al. 2020). The 982.37 mg of *C. esculenta* flower powder collected in Dschang could cover individual daily needs of magnesium as its recommended daily allowances (RDA) is 350 mg. Calcium plays major roles in the human body, notably bones and teeth solidification, muscles contraction, blood clothing, and enzymes activities (Ousaaid et al. 2022). Phosphorus contributes to the intake of calcium by bones, replication of nucleic acids, the structure of nucleoproteins, and the releasing of energy. The RDA for calcium and phosphorus is 800 mg. The Ca/P ratio greater than 1 reflects a large amount of calcium compared to phosphorus and suggests better use and bounding of calcium by bones and teeth (Tenyang et al. 2022). The sample harvested in Dschang was richer in sodium and potassium compared to that of Yabassi. Sodium is involved in maintaining osmotic pressure, blood pressure regulation, acid–base balance, and enzyme activation. Potassium plays an important role in enzyme activation, muscle contraction, osmotic regulation, membrane transport, maintenance of osmotic pressure, and acid–base balance (Manz et al. 2023). The RDA of sodium and potassium is 500 and 800 mg, respectively. Therefore, consuming 100 g and 32.79 g of powder of *C. esculenta* flowers harvested in Dschang could cover 71.34% and 100% of the RDA in sodium and potassium, respectively. Because of its low value of Na/K ratio, *C. esculenta* flower powder could be used in the regulation of blood pressure and prevention of heart attack in humans. Iron, zinc, and manganese were also present in appropriate amounts in this flower. Iron plays a key role in the human body, like oxygenation of tissue hrough transport by hemoglobin, synthesis of hormones, and cells neurotransmitters (Dama et al. 2021). Zinc contributes to taste perception, fetal development, and synthesis of genetic material. It is a cofactor that plays an essential role in the synthesis of hemoglobin, protects the integrity of erythrocytes, and reduces oxidative stress. Zinc deficiency leads to abnormal immune functions and muscle wasting. Manganese is involved in protein‐energy malnutrition, promotes activation of pyruvate carboxylase, and binding of minerals (Ayanda et al. 2019). Total carotenoids content (2201.33 μg/100 g DM and 2535.04 μg/100 g DM) varied significantly according to harvesting location. Several parameters may explain this variation, including different agro‐ecological zones, harvesting periods, and transport conditions (Mohammed et al. 2022). Carotenoids are precursors of body vitamin A. They are also powerful antioxidants, capable of preventing and fighting age‐related macular degeneration. Carotenoids protect against metabolic syndrome, cancer, photo oxidative damage to the skin and eye, and improve health and reproductive performance (Saini et al. 2022).

### 3.3 Phytochemical Screening, Total Phenolic, Flavonoid and Tannins Content of Extracts

The results of the phytochemical screening of *C. esculenta* flower are shown in Table 3. It contains alkaloids, flavonoids, triterpenes, tannins, saponins, polyphenols, and quinones as secondary metabolites. The affinity of these compounds with water could justify this result (Jounda et al. 2023). These compounds have antioxidant, antitumor, immunomodulatory, anti‐inflammatory, hypolipidemic, hypoglycemic, hypotensive, and antimicrobial properties (Vo et al. 2023). Total polyphenols, flavonoids, and tannins contents in the aqueous extracts of *C. esculenta* flowers are also shown in Table 3. The contents of these secondary metabolites vary significantly (*p <* 0.05) from one locality to another. Polyphenols are higher in the aqueous extract of flowers collected in Dschang. Values were higher than those obtained by Quinche et al. (2021), Vo et al. (2023) on the aqueous extracts of the tubers of *C. esculenta* (200 mg of EAG/100 g of dry extract) and the bark of *Garcinia mangostana* (7.5 mg EAG/g dry extract) respectively. The flavonoids are more concentrated in the aqueous extract of the flowers collected in Yabassi. Jaradat et al. (2020) and Mba et al. (2022) found flavonoids in the aqueous and hydroethanolic extracts of leaves of *Helichrysum sanguineum* (12.32 mg of QE/g of extract) and leaves of *Psychotria densinervia* (23.43 mg EC/g of extract) respectively. The flowers collected in Dschang have a higher tannin content compared to those collected from Yabassi. According to work done by Oulai et al. (2019), geographical location, climatic conditions, harvest period, and season could influence the secondary metabolite composition of a given sample. Our findings are higher compared to those obtained by Jaradat et al. (2020) on aqueous extracts of *Helichrysum sanguineum* (0.75 mg of CAE/g of extract) and Jounda et al. (2023) on aqueous extracts of *Ceiba pentandra* fruits (1.31 μg EC/mL).

**TABLE 3:** Phytochemical screening of aqueous extracts of *Colocasia esculenta* flowers.

Metabolites	Qualitative		Quantitative	
	YAq	DAq	YAq	DAq
Alkaloids	+	+		
Quinones	+	+		
Anthocyanin	+	+		
Coumarines	−	−		
Triterpene	+	+		
Saponine	+	+		
Tannins (mg EC/g of extracts)	+	+	1.83 ± 0.08^a^	2.54 ± 0.20^b^
Flavonoids (mg EQ/g of extracts)	+	+	50.43 ± 1.21^b^	39.54 ± 0.73^a^
Total Polyphenols (mg EAG/g of extracts)	+	+	32.81 ± 2.03^a^	41.51 ± 1.42^b^

*Note:* Values in line with different letters are significantly (*p* < 0.05) different. The values are the mean ± standard deviation (*n* = 3).

Abbreviations: (−), absent; (+), present; DAq, aqueous extract of *Colocasia esculenta* flowers harvested in Dschang; mg EAG/g of extract, milliequivalent of gallic acid per gram of extract; mg EC/g of extract, milliequivalent of catechin per gram of extract; mg EQ/g of extract, milliequivalent of quercetin per gram of extract; YAq, aqueous extract of *C. esculenta* flowers harvested in Yabassi.

### 3.4 Change in Body Weight and Relative Weights of Some Organs

Table 4 shows the weight changes of rats and the relative weight of some target organs. Weight gain varies significantly (*p <* 0.05) between Group 4 and Group 3. Treatment with *C. esculenta* flowers significantly increases weight gain in rats of Group 4 and Group 5 compared to Group 2. It becomes obvious that treatment based on *C. esculenta* flower powder promotes weight gain in rats. This is probably due to the quality of the nutrients contained in these flowers (Nwaogwugwu et al. 2020). The relative weight of the heart, spleen, and lungs did not vary significantly (*p >* 0.05) between the groups relative weight provides information on the growth of an organ compared to the whole organism (Nchoutpouen et al. 2020). Values of this parameter decreased significantly (*p <* 0.05) for the liver and kidneys in Group 2 compared to the other treatments. It appeared that phenylhydrazine has effects on the liver and kidneys. Different results were obtained by Shahenda and Jehan (2018), Monojit et al. (2022) who showed that intraperitoneal injection of PHZ induces hepatosplenomegaly in rats and mice, respectively. The relative weight of the target organs was not affected (*p <* 0.05) in rats treated with *C. esculenta* flower powders compared to Groups 1 and 3. *C. esculenta* powder is therefore nontoxic for these organs.

**TABLE 4:** Body weight and relative weight of some organs of rats in different groups.

	Group 1	Group 2	Group 3	Group 4	Group 5
IBW	176.20 ± 3.88^a^	181.40 ± 4.04^a^	180.60 ± 2.99^a^	178.00 ± 5.05^a^	182.00 ± 3.71^a^
FBW	180.1 ± 5.89^a^	182.9 ± 5.45^a^	187.80 ± 8.52^ab^	203.00 ± 9.01^b^	192.88 ± 7.46^ab^
WG	3.90 ± 1.05^b^	1.50 ± 0.63^a^	7.20 ± 3.58^c^	25.00 ± 5.16^d^	10.88 ± 3.64^c^
Relative weight					
Heart	0.28 ± 0.04^a^	0.29 ± 0.02^a^	0.29 ± 0.03^a^	0.31 ± 0.05^a^	0.29 ± 0.04^a^
Kidney	0.55 ± 0.03^a^	0.29 ± 0.03^b^	0.54 ± 0.05^a^	0.59 ± 0.05^a^	0.55 ± 0.05^a^
Liver	2.60 ± 0.18^a^	2.30 ± 0.25^b^	2.54 ± 0.18^a^	2.71 ± 0.32^a^	2.55 ± 0.27^a^
Spleen	0.31 ± 0.10^a^	0.26 ± 0.03^a^	0.22 ± 0.02^a^	0.30 ± 0.08^a^	0.29 ± 0.08^a^
Lung	0.60 ± 0.07^a^	0.73 ± 0.12^a^	0.58 ± 0.03^a^	0.63 ± 0.09^a^	0.71 ± 0.12^a^

*Note:* The values are the mean ± standard deviation (*n* = 5). Values in same line with different letters are significantly (*p* < 0.05) different.

Abbreviations: FBW, final body weight; IBW, initial body weight; T1, normal rats fed RSL; T2, RSL‐fed untreated anemic rats; T3, anemic rats fed RSL and treated with Apfer (5 mg/kg); T4, anemic rats fed RSL supplemented with 5% *Colocasia esculenta* powder harvested at Dschang; T5, anemic rats fed RSL supplemented with 5% *C. esculenta* powder harvested in Yabassi; WG, weightht gain.

### 3.5 Effects of *Colocasia esculenta* Flowers Powders on Erythrocyte Parameters

Table 5 shows the variations in some erythrocyte parameters. Injection of phenylhydrazine (PHZ) significantly (*p <* 0.05) decreased hematocrit, red blood cells, hemoglobin, lymphocytes, white blood cells, platelets, mean corpuscular hemoglobin concentration (MCHC), and mean corpuscular hemoglobin level (MCHL) in Groups 2 to 5 compared to Group 1 on Day 2. However, there was a significant variation (*p <* 0.05) in mean corpuscular volume (MCV) following PHZ injection in Groups 2 to 5 compared to Group 1. Similar results were reported by Bai et al. (2022), Ousaaid et al. (2022), and Shirani et al. (2023) after injection of PHZ in rats and mice. Erythrocyte hemolysis is explained by its sensitivity to oxidative damage due to the inability of cells to reduce NADP to NADPH in the normal way (Gammal et al. 2023). Inhibition of glucose‐6‐phosphate dehydrogenase (G6PD) by PHZ, which prevents the release of NADPH via the pentose phosphate pathway, is thought to promote fragility of the erythrocyte membrane (Gammal et al. 2023). PHZ induces changes in the erythrocyte membrane by oxidative denaturation of hemoglobin, leading to the alteration of hemoglobin known as ‘Heinz bodies’, which reduces the lifespan of erythrocytes (Monojit et al. 2022). The appearance of micronucleate, polychromatic, and hypochromatic erythrocytes could explain the increase in MCV (Qin et al. 2022). Treatment with *Colocassia esculenta* powder significantly (*p <* 0.05) increased all blood parameters in Groups 3 to 5 compared with Group 2 on Days 16 and 23. Similar results were obtained by Ufelle et al. (2018) who reported that the administration of methanolic extracts of *C. esculenta* leaves increased hemoglobin, red blood cell, and hematocrit levels in anemic rats. Sulistiani et al. (2020) showed that the administration of aqueous extracts of *C. esculenta* leaves to anemic and diabetic rats increased hemoglobin, red blood cell, and hematocrit levels in anemic rats. The increase in hemoglobin, red blood cell, and hematocrit levels reflects improvement in hematopoiesis due to the supply of proteins, secondary metabolites, minerals, and vitamins in the flowers (Ouoba et al. 2022). Amino acids, zinc, and iron are involved in hemoglobin synthesis, as well as the synthesis and maturation of red blood cells (Tuem et al. 2022). Hemoglobin is important for the transfer of respiratory gases. So, an abnormal drop in hemoglobin content would alter oxygen supplementation of organs (Shahenda and Jehan 2018). Thus, the consumption of *C. esculenta* flowers leads to hemoglobin balance. Consumption of *C. esculenta* flower powder significantly (*p <* 0.05) enhances white blood cells, lymphocytes, platelets, MCHC, and MCHL in anemic rats compared to untreated anemic rats. Nwaogwugwu et al. (2020) revealed that diabetic and anemic rats treated with aqueous extracts of *C. esculenta* stems had a significant increase in white blood cells, lymphocytes, and blood platelets. The increase in white blood cells, inflammation, or necrosis reactions occurred as an activation of the defense mechanism (Shirani et al. 2023). A high level of blood platelets promotes blood clotting, while a low level can cause prolonged bleeding, ecchymosis, or thrombocytopenia. Supplementation with *C. esculenta* flower powders significantly increased MCHC and MCHL in rats in Groups 4 and 5 compared to Group 2. Nwaogwugwu et al. (2020) showed that diabetic and anemic rats treated with aqueous extracts of *C. esculenta* stems had a significant increase in MCHC. Clinical analysis of MCHC and MCHL is generally used to discriminate anemia types. Low and high MCHL levels suggest hypochromia and hyperchromia, respectively (Massohe et al. 2023). Whereas a low or high MCHF suggests hypochromic or macrocytic anemia. During this treatment phase, the amount of GMV significantly (*p <* 0.05) decreased in Groups 3 to 5 compared to Group 2. Low or high MCV indicates microcytic or macrocytic anemia, respectively (Gammal et al. 2023). This result showed that the consumption of *C. esculenta* flower powder stimulates the immune system and does not induce microcytic or macrocytic anemia. Group 5 had a significant (*p <* 0.05) high values of red blood cells and platelets and low values of MCV compared to Group 3. This difference is due to the significant content of organic and mineral compounds in *C. esculenta* flowers (sugar, vitamins, amino acids, iron and other minerals) compared with the reference drug used (Apfer), which contains iron, folic acid, and vitamin B_12_ (Ouoba et al. 2022).

**TABLE 5:** Erythrocyte parameters.

Treatments	Day 0	Day 2	Day 9	Day 16	Day 23
Hematocrit (%)					
Group 1	53.00 ± 5.83^abα^	56.14 ± 2.35^bα^	53.70 ± 4.96^bα^	53.80 ± 3.03^bα^	55.40 ± 7.57^bα^
Group 2	48.70 ± 5.19^aβ^	37.20 ± 2.19^aα^	44.80 ± 1.48^aβ^	43.25 ± 2.50^aβ^	45.70 ± 2.97^aβ^
Group 3	55.43 ± 1.34^bβ^	39.22 ± 7.69^aα^	54.20 ± 5.54^bcβ^	66.50 ± 6.40^cγ^	54.17 ± 4.91^bβ^
Group 4	56.80 ± 3.03^bβ^	36.70 ± 5.54^aα^	57.20 ± 5.76^cβ^	55.40 ± 5.55^bβ^	59.00 ± 8.37^bβ^
Group 5	51.90 ± 5.34^abβ^	40.60 ± 1.14^aα^	48.20 ± 3.58^abβ^	75.00 ± 8.57^dγ^	55.20 ± 4.38^bβ^
Red blood cells (10^6^/mm^3^)					
Group 1	5.18 ± 0.45^aα^	5.76 ± 0.71^bα^	5.13 ± 0.28^bα^	5.71 ± 0.49^bα^	5.00 ± 0.59^bα^
Group 2	5.30 ± 0.71^aβ^	4.10 ± 0.57^aα^	4.08 ± 0.36^aα^	4.08 ± 0.24^aα^	4.27 ± 0.16^aα^
Group 3	5.16 ± 0.90^aβ^	3.88 ± 0.29^aα^	4.38 ± 0.36^aαβ^	5.07 ± 0.74^bβ^	5.47 ± 0.15^bβ^
Group 4	5.42 ± 0.63^aβ^	3.71 ± 0.56^aα^	4.12 ± 0.16^aα^	5.21 ± 0.42^bβ^	5.84 ± 0.77^bcβ^
Group 5	5.36 ± 0.47^aβγ^	3.57 ± 0.62^aα^	4.30 ± 0.26^aα^	4.98 ± 0.31^bβ^	5.96 ± 0.26^cγ^
Hemoglobin (g/dL)					
Group 1	10.74 ± 1.16^aα^	10.83 ± 0.17^cα^	10.62 ± 2.29^bα^	11.39 ± 0.41^bα^	11.46 ± 1.07^bα^
Group 2	11.58 ± 0.49^aβ^	6.99 ± 1.39^aα^	6.29 ± 0.69^aα^	6.38 ± 0.70^aα^	6.79 ± 1.01^aα^
Group 3	11.05 ± 0.61^aβ^	9.02 ± 0.31^bα^	10.32 ± 1.54^bαβ^	11.51 ± 0.67^bβ^	11.85 ± 0.58^bβ^
Group 4	10.27 ± 1.35^aβ^	8.12 ± 0.55^bα^	9.92 ± 1.88^bβ^	11.82 ± 0.71^bβ^	11.34 ± 0.94^bβ^
Group 5	10.52 ± 1.75^aβ^	7.43 ± 0.79^aα^	10.95 ± 2.34^bβ^	11.78 ± 0.45^bβ^	10.76 ± 1.13^bβ^
White blood cells (10^3^/mm^3^)					
Group 1	5.88 ± 0.60^aα^	5.62 ± 0.35^cα^	5.72 ± 0.39^dα^	6.86 ± 0.32^cβ^	5.09 ± 0.55^abα^
Group 2	5.80 ± 0.21^aβ^	3.54 ± 0.43^aα^	3.80 ± 0.37^aα^	4.36 ± 0.35^aαβ^	4.69 ± 0.38^aβγ^
Group 3	5.69 ± 0.20^aβγ^	4.27 ± 0.13^bα^	4.98 ± 0.11^cβ^	5.65 ± 0.34^bγ^	5.78 ± 0.31^bγ^
Group 4	5.68 ± 0.89^aβ^	4.41 ± 0.13^bα^	4.26 ± 0.38^bα^	5.52 ± 0.61^bβ^	5.26 ± 0.26^abβ^
Group 5	5.15 ± 0.60^aβ^	4.22 ± 0.02^bα^	4.23 ± 0.32^bα^	5.33 ± 0.59^bβ^	5.71 ± 0.28^bβ^
Lymphocytes(10^3^/μL)					
Group 1	0.57 ± 0.05^aα^	0.60 ± 0.04^bα^	0.59 ± 0.03^cα^	0.59 ± 0.04^bα^	0.55 ± 0.03^aα^
Group 2	0.57 ± 0.03^aβ^	0.45 ± 0.04^aα^	0.47 ± 0.03^aα^	0.50 ± 0.04^aαβ^	0.52 ± 0.05^aαβ^
Group 3	0.58 ± 0.02^aβ^	0.47 ± 0.04^aα^	0.53 ± 0.02^bαβ^	0.57 ± 0.03^abβ^	0.54 ± 0.08^aαβ^
Group 4	0.57 ± 0.02^aβ^	0.46 ± 0.03^aα^	0.58 ± 0.04^bcβ^	0.56 ± 0.07^abαβ^	0.56 ± 0.04^aβ^
Group 5	0.55 ± 0.07^aαβ^	0.48 ± 0.05^aα^	0.62 ± 0.05^cβ^	0.62 ± 0.04^bβ^	0.57 ± 0.02^aβ^
Blood platelets (10^3^/mm^3^)					
Group 1	156.80 ± 7.60^aα^	152.80 ± 4.87^cα^	155.60 ± 1.14^bα^	157.60 ± 4.88^bα^	154.40 ± 1.52^bα^
Group 2	156.90 ± 3.01^aδ^	136.80 ± 0.45^aα^	140.00 ± 2.82^aβ^	145.82 ± 3.03^aβγ^	147.75 ± 3.68^aγ^
Group 3	155.60 ± 3.97^aβγ^	144.38 ± 2.24^bα^	151.80 ± 1.92^bβ^	156.75 ± 2.63^bγ^	159.67 ± 3.17^bγ^
Group 4	150.40 ± 8.99^aαβ^	144.08 ± 1.90^bα^	151.40 ± 7.37^bαβ^	153.20 ± 5.57^bβ^	155.00 ± 2.89^bβ^
Group 5	151.22 ± 6.20^aβ^	143.40 ± 2.07^bα^	147.00 ± 5.83^abαβ^	151.10 ± 5.43^abβ^	186.00 ± 4.12^cγ^
VGM (μm^3^)					
Group 1	52.20 ± 7.26^aα^	59.00 ± 2.55^aα^	54.60 ± 5.81^aα^	53.38 ± 1.10^aα^	54.87 ± 2.96^aα^
Group 2	51.24 ± 6.93^aα^	79.67 ± 5.88^bγ^	80.80 ± 6.83^cdγ^	71.50 ± 1.76^cβ^	75.76 ± 5.11^dβγ^
Group 3	55.06 ± 3.18^aα^	82.60 ± 1.10^bγ^	85.80 ± 1.30^dδ^	63.88 ± 2.75^bβ^	66.33 ± 2.37^cβ^
Group 4	62.60 ± 8.26^aαβ^	81.60 ± 2.30^bγ^	69.60 ± 4.02^bβ^	56.60 ± 2.61^aα^	59.92 ± 3.04^abα^
Group 5	57.30 ± 1.44^aα^	82.80 ± 1.10^bγ^	81.20 ± 2.68^cγ^	63.00 ± 2.29^bβ^	60.28 ± 0.44^bβ^
MCH (g/dL)					
Group 1	48.20 ± 5.76^aα^	48.00 ± 3.24^cα^	48.70 ± 3.87^cα^	47.00 ± 8.25^cα^	52.60 ± 1.82^bα^
Group 2	58.60 ± 1.34^bγ^	32.20 ± 3.27^bα^	33.20 ± 2.45^aα^	35.63 ± 1.50^aα^	41.38 ± 7.82^aβ^
Group 3	56.80 ± 5.68^abγ^	39.78 ± 4.55^bα^	41.40 ± 5.50^bcαβ^	46.00 ± 1.69^cβ^	57.83 ± 3.18^cγ^
Group 4	49.60 ± 6.39^aβγ^	33.00 ± 2.74^bα^	40.21 ± 3.67^bβ^	44.40 ± 2.64^cβ^	52.60 ± 2.61^bγ^
Group 5	57.50 ± 4.33^abδ^	27.20 ± 1.79^aα^	43.00 ± 5.66^bcγ^	38.98 ± 1.05^bβ^	57.08 ± 2.8^cδ^
MCHC (pg)					
Group 1	58.40 ± 6.77^aαβ^	55.00 ± 4.74^bαβ^	54.00 ± 1.73^cα^	59.40 ± 2.30^cβ^	53.00 ± 1.41^bα^
Group 2	53.60 ± 3.34^aγ^	40.00 ± 4.80^aα^	38.60 ± 4.45^abα^	41.50 ± 2.76^aα^	47.25 ± 0.40^aβ^
Group 3	56.80 ± 2.69^aγ^	42.38 ± 1.52^aα^	48.60 ± 0.65^bβ^	48.25 ± 1.50^bβ^	57.67 ± 2.89^cγ^
Group 4	52.00 ± 4.24^aβγ^	43.04 ± 2.13^aα^	42.20 ± 4.92^aα^	49.80 ± 1.22^bβ^	52.60 ± 1.61^bγ^
Group 5	53.50 ± 4.87^aβγ^	40.44 ± 0.89^aα^	46.20 ± 5.95^abαβ^	49.60 ± 0.89^bβ^	57.08 ± 2.85^cγ^

*Note:* Columns values with different letters are significantly (*p* < 0.05) different. Values in the same line with different symbols are significantly (*p* < 0.05) different. The values are the mean ± standard deviation (*n* = 5). Group 1, normal rats fed RSL; Group 2, RSL‐fed untreated anemic rats; Group 3, anemic rats fed RSL and treated with Apfer (5 mg/kg); Group 4, anemic rats fed RSL supplemented with 5% *Colocasia esculenta* flower powder harvested in Dschang; Group 5, anemic rats fed RSL supplemented with 5% *C. esculenta* flower powder harvested in Yabassi.

Abbreviations: MCH, mean corpuscular hemoglobin concentration; MCHC, mean corpuscular hemoglobin content; VGM, mean globular volume.

### 3.6 Effects of *Colocasia esculenta* Flower Powder on Some Biochemical Markers

Some biochemical and stress markers were analyzed (Table 6). Intraperitoneal injection of phenylhydrazine (PHZ) significantly (*p* < 0.05) increased TG, LDL, ASAT, ALAT, urea, creatinine, PAL, and γGT in Group 2 compared to Group 1. PHZ appeared as a hypertriglyceridaemic, hepatotoxic, and renotoxic drug. Shahenda and Jehan (2018) showed that injection of PHZ in rats resulted in increased transaminase and PAL activities compared to normal rats. The release of cellular contents such as lipids following erythrocytes haemolysis could explain the hypertriglyceridaemia state (Qin et al. 2022). The kidneys and liver are involved in body homeostasis and can be affected by various drugs or chemicals, including xenobiotics (Gammal et al. 2023). Increase of ASAT, ALAT, PAL, and γGT activities, urea, and creatinine levels would reflect liver and kidney damage (Massohe et al. 2023). Treatment with Apfer significantly (*p <* 0.05) reduced TG, LDL, creatinine, urea, transaminase (ASAT, ALAT), PAL, and γGT activity in Group 3 compared to Group 2. This result reveals the hepatorenoprotective effect of Apfer. Apfer contains iron, vitamin B12, and folic acid, which are necessary substances for hematopoiesis (Ouoba et al. 2022). Supplementation with *C. esculenta* flower powder significantly (*p <* 0.05) reduced TC (27.51%), TG (32.13%), LDL (46.30%), ASAT (65.39%), ALAT (22.90%), PAL (13.77%), γGT (18.57%), creatinine (66.73%) and urea (46.76%) levels in Group 4 compared to Group 2. In Group 5, TC, TG, LDL, creatinine, urea, and the activity of ASAT, ALAT, PAL, and γGT by 25.32%, 43.31%, 79.10%, 69.02%, 42.85%, 60.79%, 56.90%, 18.14%, and 29.94%, respectively, significantly (*p <* 0.05) decreased compared to Group 2. HDL‐c increased significantly (*p <* 0.05) in rats of Groups 4 and 5 compared to Group 2. In comparison to Group 3, we observed a significant (*p <* 0.05) reduction of TC, TG, LDL, AST, γGT and creatinine in Groups 4 and 5. Compared to Group 4, HDL increased and LDL decreased significantly (*p <* 0.05) in Group 5. These results suggest that *C. esculenta* flower powders have a hypolipidemic effect and protect the liver and kidneys from injuries. This finding was also confirmed by Azubike et al. (2017). Eneh et al. (2020) found that *C. esculenta* leaf extracts reduced blood lipids in rats. The reduction in lipid profile, liver, and kidneys markers could be attributed to the fibers and phytochemicals that improve the inflammatory response (Kumar et al. 2023). These metabolites inhibit Series 2 eicosanoids, which are involved in inflammatory processes (Ashish and Jay 2023). Fibers reduce the absorption of cholesterol in the intestine, thereby reducing its blood levels (Tuem et al. 2022). Flavonoids stimulate the activity of lecithin cholesterol acyltransferase (LCAT) and 7α‐hydrolase, involved in the pathway of bile acids synthesis. They prevent oxidation of low‐density lipoprotein, inhibit lanosterol synthase, which is involved in cholesterol synthesis. Flavonoids reduce hypertension and cardiovascular diseases through their vasodilatory and platelet aggregation inhibitory effects (Jounda et al. 2023). Terpenoids have antioxidant potential, which can prevent tissue failure and heart attack. Saponins inhibit acetyl‐CoA carboxylase, which is involved in fatty acid synthesis, and prevent heart attack through their antioxidant potential (Jyothi and Srinivas 2019).

**TABLE 6:** Biochemical markers (lipid profile, liver toxicity and enzymes inhibitions) and oxidative stress parameters during experimentation.

Parameters	Group 1	Group 2	Group 3	Group 4	Group 5
TC (mg/dL)	135.07 ± 5.76^b^	141.91 ± 10.71^b^	131.69 ± 3.40^b^	102.86 ± 5.99^a^	105.97 ± 4.77^a^
TG (mg/dL)	150.80 ± 5.69^c^	161.95 ± 3.37^d^	132.21 ± 6.02^b^	109.91 ± 11.42^a^	106.37 ± 10.72^a^
HDL (mg/dL)	50.00 ± 6.73^c^	25.25 ± 2.47^a^	39.70 ± 5.62^b^	37.07 ± 3.14^b^	67.78 ± 1.31^d^
LDL (mg/dL)	52.12 ± 1.93^c^	85.39 ± 6.95^e^	62.91 ± 4.70^d^	45.85 ± 4.31^b^	17.84 ± 4.21^a^
Proteinuria (g/L)	23.05 ± 2.34^a^	38.29 ± 1.86^c^	35.12 ± 1.56^c^	36.99 ± 2.77^c^	31.23 ± 1.49^b^
ALAT (UI/L)	14.84 ± 0.82^b^	36.20 ± 2.46^d^	6.46 ± 1.17^a^	27.91 ± 2.44^c^	15.60 ± 2.71^b^
ASAT (UI/L)	13.91 ± 1.73^c^	21.53 ± 2.25^d^	10.13 ± 1.21^b^	7.45 ± 0.76^a^	8.44 ± 0.92^ab^
Creatinine (mg/L)	1.46 ± 0.21^a^	4.81 ± 0.56^c^	3.03 ± 0.26^b^	1.60 ± 0.40^a^	1.49 ± 0.26^a^
Urea (mg/L)	66.67 ± 3.73^b^	128.33 ± 12.64^c^	60.00 ± 1.06^a^	72.17 ± 6.07^b^	73.33 ± 9.13^b^
PAL (UI/L)	32.34 ± 3.54^b^	42.10 ± 2.64^c^	22.48 ± 2.12^a^	36.30 ± 1.24^b^	34.46 ± 3.11^b^
γGT (UI/L)	6.37 ± 1.10^a^	9.15 ± 0.56^b^	10.13 ± 1.21^c^	7.45 ± 0.76^a^	6.41 ± 0.75^a^
SOD (unit/mg protein)	17.42 ± 1.14^b^	7.78 ± 0.34^a^	18.77 ± 0.56^b^	47.62 ± 3.33^d^	20.70 ± 0.79^c^
CAT (nM of H_2_O_2_/min/mg protein)	166.62 ± 8.14^e^	51.47 ± 2.71^a^	129.26 ± 13.00^d^	95.24 ± 7.77^c^	81.52 ± 6.11^b^
GSH (μM/mg protein)	65.04 ± 2.18^e^	21.73 ± 3.05^a^	38.51 ± 3.07^b^	48.37 ± 2.68^c^	54.31 ± 1.74^d^
TBARS (mm MDA/mg of protein)	5.40 ± 0.74^a^	18.36 ± 0.58^e^	7.68 ± 0.57^b^	13.13 ± 0.76^d^	10.14 ± 0.92^c^

*Note:* The values are the mean ± standard deviation (*n* = 5). Values in line with different letters are significantly (*p* < 0.05) different. Group 1, normal rats fed RSL; Group 2, RSL‐fed untreated anemic rats; Group 3, anemic rats fed RSL and treated with Apfer (5 mg/kg); Group 4, anemic rats fed RSL supplemented with 5% *Colocasia esculenta* flower powder harvested in Dschang; Group 5, anemic rats fed RSL supplemented with 5% *C. esculenta* flower powder harvested in Yabassi.

Abbreviations: γGT, Gamma glutamyl transferase; ALAT, alanine aminotransferase; ALP, phosphatase alcaline; ASAT, aspartate aminotransferase; CAT, catalase; HDL, high density lipoprotein; LDL, low density lipoprotein; MDA, malondialdehyd; SOD, superoxide dismutase; TBARS, thiobarbituric acid reactive substances; TC, total cholesterol; TG, triglycerides.

### 3.7 Effects of *Colocasia esculenta* Flower Powders on Markers of Oxidative Stress

Table 6 shows the value of markers of oxidative stress of the different groups of rats. Intraperitoneal injection of phenylhydrazine (PHZ) significantly (*p <* 0.05) increased TBARS concentration and decreased glutathione concentration, SOD and CAT activities in Group 2 compared to the other groups. Similar results were observed by Monojit et al. (2022) and Qin et al. (2022) in mice. PHZ destroys blood cells, notably red blood cells, through the overproduction of reactive oxygen species (ROS) that denature hemoglobin, converting oxyhemoglobin into a powerful oxidant involved in the aging and apoptosis of erythrocytes (Bai et al. 2022). The inability of erythrocytes to protect sulfhydryl groups against oxidative damage generated by PHZ could explain the decrease in thiol functions. The increase in TBARS is thought to reflect increased lipid peroxidation following oxidation of membrane phospholipids by PHZ (Ousaaid et al. 2022). Treatment with the reference drug Apfer significantly (*p <* 0.05) reduced TBARS concentration and increased glutathione concentration, SOD and CAT activities in Group 3 compared to Group 2. Treatment with *C. esculenta* flower powders significantly (*p <* 0.05) increased SOD and CAT activity and GSH levels. However, it significantly decreased (*p* < 0.05) TBARS levels in Groups 4 and 5 compared to Group 2 rats. We also found that *C. esculenta* flowers increased (*p <* 0.05) GSH levels and SOD activity in Groups 4 and 5 more than in Group 3. SOD and CAT activities significantly (*p <* 0.05) increased in Group 4 compared to Group 5. However, this group had significant (*p <* 0.05) low levels of TBARS and high concentrations of GSH. Oxidative stress is thought to be involved in the onset of hemolytic anemia. G6PD‐deficient erythrocytes are sensitive to free radicals generated by certain drugs (Gammal et al. 2023). The secondary metabolites present in *C. esculenta* flowers have antioxidant properties, trapping and capturing the free radicals (Jyothi and Srinivas 2019). Hence, their usefulness in combating anemia through their antioxidant properties.

## 4 Conclusion

The aim of this study was to assess the nutritional potential of *C. esculenta* flowers and their effects on anemia and oxidative stress in Wistar rats. The results showed that *C. esculenta* flowers contain significant levels of macronutrients and minerals. The leaves harvested in Dschang were richer in proteins and minerals (Ca, Mg, Fe, Na, K and P), while those from Yabassi contained more carbohydrates, fiber, and ash. The consumption of leaves from Yabassi improved the weight of the animals the most. Supplements based on *C. esculenta* flower powders restored hematocrit, hemoglobin, and red blood cell levels in anemic rats, with a greater potential for expression in animals fed Dschang leaves. They also helped improve hepatorenal function by reducing GT, PAL, urea, and creatinine. *C. esculenta* flowers are also a natural source of antioxidants responsible for increasing the activity of antioxidant enzymes (SOD and CAT) and inhibiting the synthesis of MDA, a marker of lipid peroxidation. Treatment with *C. esculenta* flower powders helped to reduce the level of oxidative stress in anemic rats. *C. esculenta* flowers have a good nutritional profile for use in the treatment of anemia and oxidative stress. Further studies will be needed to make this work more reliable.

## 5 Limitations

1. In the study, a single dose was used for treatment instead of multiple doses in this case, three doses.
2. The biochemical mechanism of action of the flowers or their components was not elucidated.
3. Assess the food and energy intake of the rats during treatment.
4. Assess the influence of taro flowers consumption on the immune response of the *Wistar* rats.

## Author Contributions

**Josée Rebecca Nombo:** conceptualization (equal), formal analysis (equal), investigation (equal), methodology (equal), resources (equal), writing – original draft (equal). **Armel Junior Mbock:** conceptualization (equal), data curation (equal), formal analysis (equal), methodology (equal), visualization (equal), writing – review and editing (equal). **K. J. C. Manz:** conceptualization (equal), data curation (equal), formal analysis (equal), investigation (equal), methodology (equal), resources (equal), software (equal), writing – original draft (equal). **Tene Stephano Tambo:** data curation (equal), formal analysis (equal), methodology (equal), writing – original draft (equal). **Misse Jethro Roland Ngangue Ekwalla:** formal analysis (equal), methodology (equal), writing – original draft (equal). **Valery Jean François Nsoga:** data curation (equal), investigation (equal), resources (equal), writing – review and editing (equal). **Mathieu Ndomou:** conceptualization (equal), project administration (supporting), supervision (supporting), writing – review and editing (equal).

## Ethics Statement

This work required the intervention of the animal's subjects, and all the experiments were conducted in accordance with the internationally accepted guidelines for experimental animals use, and the study was approved by the University of Douala Institutional Ethics Committee (No. 2304 CEI‐Udo/05/2020/T).

## Conflicts of Interest

The authors declare that this work is original and has not been published or submitted for publication in any other journal. They also declare that they have no financial, technical, or moral conflicts of interest with any third party that would prevent publication of this article.

## Data Availability

Data will be made available on request.
